# Acrodermatitis Enteropathica: A Rare Case With Lifelong Implications

**DOI:** 10.7759/cureus.37783

**Published:** 2023-04-18

**Authors:** Muhannad M Alwadany, Abdullah F Al Wadani, Fatimah H Almarri, Hadi S Alyami, Muhammad A Al-Subaie

**Affiliations:** 1 General Practice, King Faisal University, Hofuf, SAU; 2 Dermatology, King Fahd Hospital of the University, Khobar, SAU

**Keywords:** case report, dermatitis, zinc deficiency, genetic disorder, acrodermatitis enteropathica

## Abstract

Acrodermatitis enteropathica is a rare genetic disorder caused by a defect in intestinal zinc absorption, resulting in zinc deficiency and various clinical manifestations, including dermatitis, diarrhea, alopecia, and nail abnormalities. Here we present the case of a 10-year-old male child with diarrhea, and abdominal pain for several months who was diagnosed with acrodermatitis enteropathica confirmed by low serum zinc levels. The child had multiple erythematous, scaly, and crusted lesions on the hands and elbows, which resolved after starting oral zinc sulfate supplementation (10 mg/kg/day) in three divided doses. The patient's serum zinc levels normalized (1.0 µg/mL), and the skin lesions completely resolved after six months of follow-up with a regular zinc-rich diet and gradual reduction of zinc sulfate dosage to a maintenance level (2-4 mg/kg/day). This case report emphasizes the importance of timely diagnosis and treatment of acrodermatitis enteropathica to prevent the harmful consequences of zinc deficiency and highlights the need for healthcare providers to consider this disorder in children presenting with skin lesions and diarrhea, particularly those with a positive family history or consanguinity.

## Introduction

Acrodermatitis enteropathica is a rare genetic disorder that was first described by Danbolt and Closs in 1942 [1]. It is caused by a defect in intestinal zinc absorption, which leads to decreased levels of zinc in serum and tissues, resulting in a variety of clinical manifestations. The name "acrodermatitis enteropathica" reflects the fact that the disorder is associated with intestinal malabsorption and that the skin lesions are typically located on the extremities [1].

The genetic basis of acrodermatitis enteropathica was elucidated when the SLC39A4 gene was identified as the causative gene. The SLC39A4 gene encodes for the zinc transporter protein ZIP4, which is responsible for the uptake of zinc from the diet in the small intestine. Mutations in the SLC39A4 gene result in impaired zinc absorption, leading to decreased levels of zinc in serum and tissues [2]. Acrodermatitis enteropathica can be inherited in an autosomal recessive manner or can occur sporadically due to de novo mutations, meaning that the mutation arises spontaneously during gametogenesis or early embryonic development.

## Case presentation

A 10-year-old male child was referred to the dermatology clinic for evaluation of skin lesions on his extremities. The child's parents reported that he had a poor appetite and had been experiencing diarrhea and abdominal pain for the past several months. The child's developmental milestones were age-appropriate, and there was no known family history of skin diseases. The patient had experienced a weight loss of four kilograms over the preceding two months, but his body mass index was 19 kg/m^2^, which is normal for his age. The patient did not mention any previous infections of his skin lesions in his medical history, and he has no history of immunodeficiency or growth retardation. Additionally, the child's parents were consanguineous.

**Figure 1:**
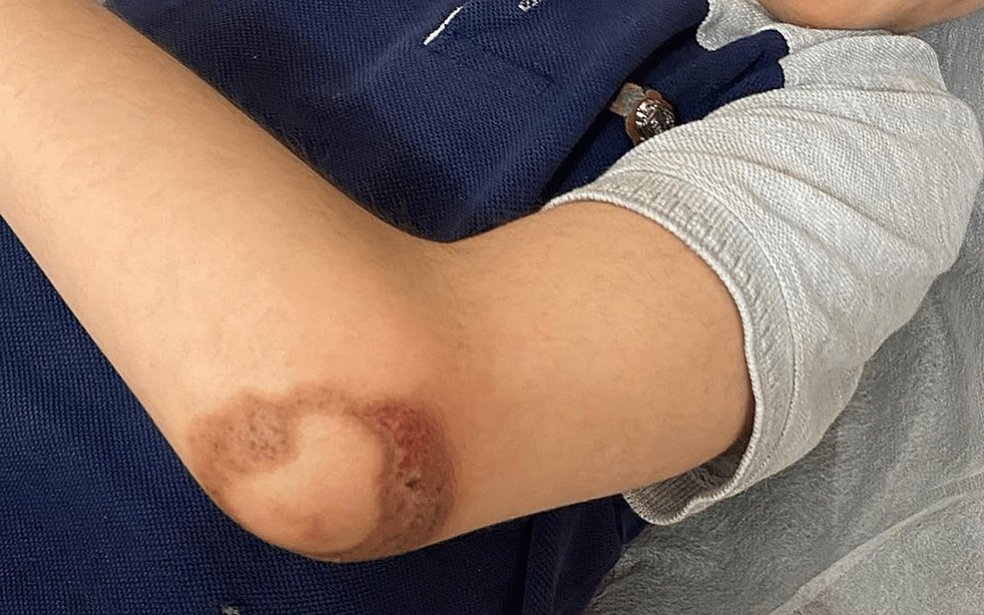
Photograph depicting an erythematous, scaly, and crusted lesion with a serpiginous appearance on the left elbow.

**Figure 2:**
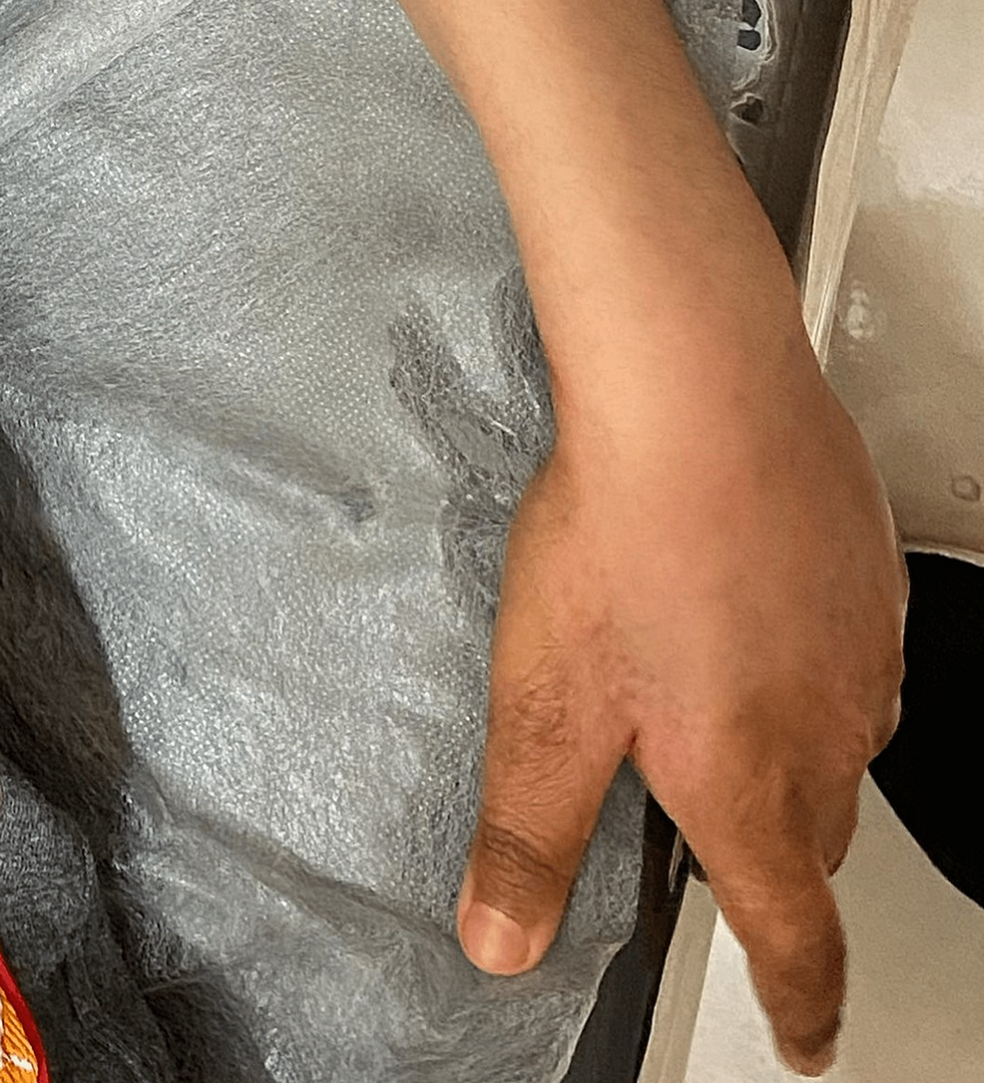
Photograph illustrating erythematous, scaly, and crusted lesions on the phalanges.

On physical examination, the patient appeared well-nourished but had multiple erythematous, scaly, and crusted lesions on the hands (mainly the phalanges) and elbow. The largest of which was seen over the left elbow. The lesions were well-demarcated, and some had a serpiginous appearance (Figure 1 and Figure 2). Additionally, the patient had no angular cheilitis or oral mucosal ulcerations. A complete skin examination did not reveal any other abnormalities. Notably, the patient did not try any treatment for the skin lesions before seeking medical attention.

Laboratory investigations showed low serum zinc levels, confirming the diagnosis of acrodermatitis enteropathica. The patient’s serum copper, iron, and other micronutrient levels were within the normal range. Further workup included a complete blood count, liver function tests, and serum electrolyte levels, which were all normal (Table 1).

**Table 1:** Results of laboratory investigations

Laboratory investigations		Result	Normal range
Trace Elements	Serum zinc levels	0.4 µg/mL	0.7-1.5 µg/mL
	Serum copper levels	80 µg/dL	70-140 µg/dL
	Serum iron levels	80 µg/dL	40-150 µg/dL
Complete Blood Count	Hemoglobin	13 g/dL	11.5-15.5 g/dL
	White blood cell count	7.5 × 10^9^/L	4.5-11.0 × 10^9^/L
	Platelet count	300 × 10^9^/L	150-400 × 10^9^/L
Electrolytes	Sodium	140 mmol/L	135-145 mmol/L
	Potassium	4.0 mmol/L	3.5-5.0 mmol/L
	Chloride	102 mmol/L	96-106 mmol/L
	Bicarbonate	26 mmol/L	22-28 mmol/L
Liver Function Tests	Aspartate transaminase	24 IU/L	5-40 IU/L
	Alanine transaminase	22 IU/L	5-40 IU/L
	Alkaline phosphatase	140 IU/L	40-150 IU/L
	Total bilirubin	0.5 mg/dL	0.1-1.2 mg/dL
	Albumin	4.2 g/dL	3.4-5.4 g/dL

The patient was started on oral zinc sulfate supplementation (10 mg/kg/day) in three divided doses, and his symptoms improved dramatically within a few weeks. The skin lesions started resolving, and the patient’s appetite and gastrointestinal symptoms improved. The patient was followed up for six months, during which time his serum zinc levels normalized (1.0 µg/mL) and the skin lesions completely resolved.

The diagnosis of acrodermatitis enteropathica was also confirmed by the genetic study. Then, the patient’s parents were counseled on the importance of maintaining an appropriate zinc-rich diet, such as meat, eggs, and dairy products, and regular follow-up with their pediatrician. The patient’s zinc sulfate dosage was gradually reduced to a maintenance level (2-4 mg/kg/day), and he remained asymptomatic with no recurrence of skin lesions during the six-month follow-up period.

## Discussion

Acrodermatitis enteropathica is a rare genetic disorder that is characterized by a defect in intestinal zinc absorption, leading to zinc deficiency and a variety of clinical manifestations, including dermatitis, diarrhea, alopecia, and nail abnormalities. Acrodermatitis enteropathica is caused by mutations in the SLC39A4 gene, which encodes for the zinc transporter protein ZIP4 [3]. The disorder can be inherited in an autosomal recessive manner or can occur sporadically due to de novo mutations. The severity of the disease can vary widely, depending on the age of onset, the extent of zinc deficiency, and the presence of concomitant infections [3].

Our case report describes a 10-year-old boy with acrodermatitis enteropathica who presented with skin lesions and diarrhea. The patient’s serum zinc levels were found to be low, confirming the diagnosis of acrodermatitis enteropathica. Other laboratory investigations, including a complete blood count, electrolytes, and liver function tests, were within normal limits, ruling out other causes of micronutrient deficiencies and indicating that the patient did not have any significant blood, electrolyte, or liver abnormalities.

The skin lesions in acrodermatitis enteropathica are typically erythematous, scaly, and crusted and can occur in a variety of patterns, including periorificial, acral, and intertriginous [4]. The dermatitis can be complicated by secondary bacterial, fungal, or viral infections, leading to further skin damage and systemic illness [1,4]. In our case, the patient presented with acral dermatitis, which responded well to oral zinc supplementation.

Zinc is an essential micronutrient that is required for a variety of biological processes, including protein synthesis, DNA replication, and immune function. Zinc deficiency can lead to growth retardation, immune dysfunction, and increased susceptibility to infections. In acrodermatitis enteropathica, the deficiency of zinc is caused by impaired absorption of the metal in the small intestine, leading to decreased levels of zinc in serum and tissues. The severity of the deficiency can be assessed by measuring serum zinc levels, which are usually low in patients with acrodermatitis enteropathica [2].

The treatment of acrodermatitis enteropathica involves lifelong oral zinc supplementation, which can reverse the clinical manifestations of the disease and prevent further complications. The recommended dose of zinc is 1-2 mg/kg/day, divided into two or three doses [2,5]. The response to therapy is usually rapid, with improvements in skin lesions, diarrhea, and growth within a few weeks. In our case, the patient responded well to oral zinc supplementation, with complete resolution of skin lesions and diarrhea within three weeks of therapy initiation.

## Conclusions

This case report emphasizes the significance of diagnosing and treating acrodermatitis enteropathica promptly to avoid the detrimental effects of zinc deficiency. Healthcare professionals should consider acrodermatitis enteropathica in the differential diagnosis of children with skin lesions and diarrhea, especially in cases with a positive family history or consanguinity. Further research is needed to deepen our understanding of the underlying mechanisms of acrodermatitis enteropathica and to develop new treatments for this rare genetic disorder.
